# CloneFast: A simple plasmid design and construction guide for labs venturing into synthetic biology

**DOI:** 10.1016/j.xpro.2025.104025

**Published:** 2025-08-06

**Authors:** Vincent Fung, Palas Balakdas Tiwade, Owen S. Fenton

**Affiliations:** 1 Division of Pharmacoengineering and Molecular Pharmaceutics, Eshelman School of Pharmacy, University of North Carolina at Chapel Hill, Chapel Hill, NC 27599, USA

## Abstract

Traditional plasmid assembly often involves expensive or restrictive enzymatic processes. To overcome these limitations, we introduce the CloneFast guide, a streamlined approach for efficient plasmid construction. Initially, this guide advises users through bioinformatic plasmid and oligonucleotide design *in silico*. Subsequently, we outline practical experimental steps, including the introduction of phosphorothioate modifications via reusable modified oligonucleotides (CompetePCR), generation of precise sticky ends through a rapid iodine-mediated cleavage, and seamless fragment assembly into plasmids. CloneFast enables the completion of plasmid assembly within 3 days, offering versatility and accessibility to a wide range of research groups.

## Graphical abstract

**Figure:**
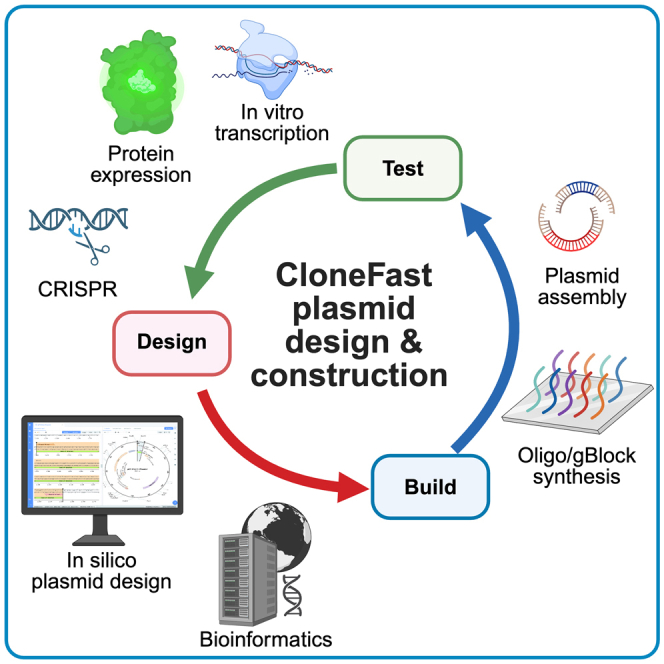

## Introduction

For complete details on the background, use, and execution of this guide, please refer to Zou et al., Ma et al., and Fung et al.^1^^,^^2^^,^^3^ Plasmids are essential tools in biotechnology, serving as vectors to introduce and manipulate genes within cells.^4^^,^^5^^,^^6^^,^^7^^,^^8^^,^^9^ Their versatile applications extend to research fields such as metabolic engineering, synthetic biology, and gene therapy.^3^^,^^10^^,^^11^^,^^12^^,^^13^ In metabolic engineering, plasmids can be used to optimize and tune microbial pathways to enhance the production of biofuels, chemicals, and pharmaceuticals.^2^^,^^14^^,^^15^^,^^16^^,^^17^^,^^18^^,^^19^ In synthetic biology, plasmids can be used to construct complex genetic circuits that enable programmable cellular behaviour.^19^^,^^20^^,^^21^^,^^22^^,^^23^^,^^24^^,^^25^^,^^26^ In gene therapy, plasmids can be used to deliver therapeutic genes to target cells or act as precursors for other therapeutic molecules, such as messenger ribonucleic acid (mRNA) and proteins, thereby offering potential cures for genetic disorders.^27^^,^^28^^,^^29^^,^^30^^,^^31^^,^^32^^,^^33^^,^^34^^,^^35^^,^^36^^,^^37^^,^^38^^,^^39^^,^^40^^,^^41^^,^^42^^,^^43^^,^^44^^,^^45^^,^^46^^,^^47^^,^^48^^,^^49^^,^^50^^,^^51^^,^^52^^,^^53^^,^^54^^,^^55^^,^^56^^,^^57^^,^^58^^,^^59^^,^^60^^,^^61^^,^^62^^,^^63^^,^^64^^,^^65^^,^^66^^,^^67^^,^^68^^,^^69^ Constructing customized plasmids in-house provides several significant advantages, including rapid iteration of genetic sequences, flexibility to address specific research needs, and reduced costs compared to outsourcing.^2^^,^^70^^,^^71^ This in-house capability accelerates research timelines and provides laboratories with greater control over plasmid design and modification.^2^^,^^72^^,^^73^ Despite the availability of several plasmid construction methods and standards, such as restriction-enzyme-based cloning and Gibson assembly, the expertise required for routine execution and the high cost of associated reagents present significant barriers for laboratories newly entering the field of synthetic biology.^2^^,^^74^^,^^75^^,^^76^^,^^77^^,^^78^ In response, we present a detailed guide for fundamental bioinformatics techniques to design plasmids and oligos *in silico* using Benchling, and a general plasmid design and construction workflow, designed to facilitate easy, low-cost, and rapid adoption by any laboratory.

### Use of phosphorothioate-modified oligonucleotides to construct plasmids

**Figure 1:**
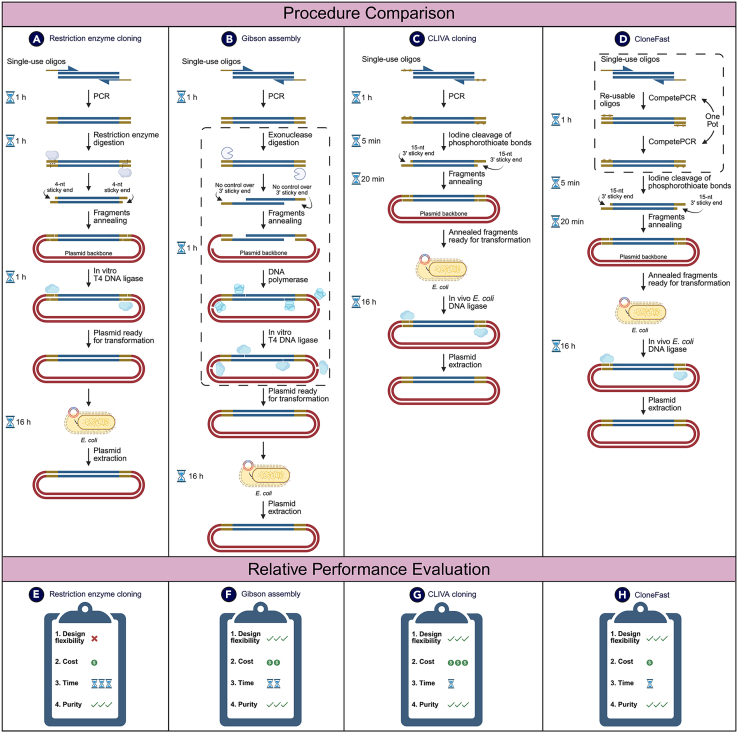
Comparison of plasmid construction procedures This figure illustrates the stepwise process for each procedure, including DNA fragment amplification via PCR; generation of sticky ends through enzymatic digestion using restriction enzymes, exonucleases, or cleavage of phosphorothioate-modified bonds via an iodine-mediated reaction; DNA assembly (either *in vitro* or *in vivo*), *E. coli* transformation; and plasmid extraction. The dotted-lined boxes in Gibson assembly and CloneFast procedures indicate that the multi-step process occurs in a one-pot fashion. The performance evaluation section compares these methods based on design flexibility, cost, time required, and product purity. (A and E) depict restriction-enzyme-based cloning, (B and F) Gibson assembly, (C and G) CLIVA cloning, and (D and H) CloneFast. Sticky end generation is accomplished differently across plasmid construction procedures. In restriction-enzyme-based cloning, a 4-nt sticky end is generated when a restriction enzyme cleaves the DNA at a specific recognition site. In Gibson assembly, an exonuclease digests the 3′ end of each DNA strand in an uncontrolled manner to create sticky ends. In CLIVA and CloneFast procedures, DNA fragments contain phosphorothioate-modified bonds introduced by PCR, which are cleaved via a 5-min iodine-mediated reaction to generate 15-nt sticky ends. CLIVA and CloneFast procedure allows *in vivo* DNA assembly due to the long, 15-nt sticky ends, unlike restriction-enzyme-based cloning and Gibson assembly. The key difference between CLIVA and CloneFast is that the phosphorothioate-modified oligos can be reused in the CloneFast procedure, which may reduce costs. CloneFast offers advantages in time efficiency and cost-effectiveness, as it does not require specific recognition sites or expensive DNA ligase and exonuclease, while maintaining high purity and flexibility.

Cross-lapping *in vitro* assembly (CLIVA) was first developed by Zou to accurately assemble seven DNA fragments encoding enzymes for isoprenoid production from the 1-deoxy-D-xylulose 5-phosphate pathway into a 21.6 kilobase (kb) plasmid.^1^ This method harnessed the highly efficient and site-specific cleavage of phosphorothioate-modified nucleotides by iodine in an ethanol solution for 5 min, generating sticky ends at both ends of the DNA fragment (Figure 1C).^1^^,^^79^^,^^80^ Two phosphorothioate modifications were introduced into the DNA fragments via phosphorothioate-modified oligos during polymerase chain reaction (PCR).^1^ The location of the phosphorothioate bonds was strategically placed in the oligos such that it would generate 15-nucleotides (15-nt) sticky ends after reacting with iodine.^1^ Because restriction enzymes were not needed to generate sticky ends, the CLIVA workflow was entirely sequence independent, providing a high degree of flexibility.^1^ However, a limitation of this workflow was that the phosphorothioate-modified oligos could not be reused for different DNA fragments, substantially contributing to the high cost.

Ma and co-authors in Zhou’s laboratory subsequently developed the guanine/thymine standard (GTS) for plasmid construction, aimed at enabling the reusability of phosphorothioate-modified oligos, thereby significantly reducing the cost of plasmid assembly.^2^ Each DNA fragment is flanked and barcoded with a pair of short, defined DNA sequences through a simple *in vitro* DNA ligase reaction.^2^ These barcode sequences are essential genetic parts that can be reused for different plasmid constructs, such as promoters, ribosomal binding sites, and terminators. The barcoded DNA fragment is subsequently amplified via PCR using reusable phosphorothioate-modified oligos specific to the barcode sequence.^2^ The authors reported that up to seven barcoded DNA fragments could be assembled into a single plasmid with an average assembly accuracy of 85.9%.^2^ However, GTS was primarily effective for DNA fragments up to 2 kb in length, which limited its application in plasmid design, as many genes exceed 2 kb and there is often a need to clone operons containing multiple genes.^2^ As a result, the CLIVA protocol would be used to construct plasmids if DNA fragments are larger than 2 kb.

To address the challenges associated with the high costs of phosphorothioate-modified oligos and the sequence length limitation of 2 kb, Fung and co-authors in Zhou’s laboratory developed a novel one-step PCR workflow called CompetePCR (Figure 1D).^3^ This workflow requires two pairs of oligos: Boligos, which are non-modified oligos that encode the barcoding sequence and the sequence that targets and amplifies the DNA fragment (serving as low-cost, customizable parts), and Aoligos, which are phosphorothioate-modified oligos that encode the barcoding sequence as described in the GTS protocol (serving as reusable parts).^3^ The success of the workflow depended on optimizing the ratio of Boligos to Aoligos in the PCR mix.^3^ Initially, the minimal amount of Boligos in the PCR mixture introduces the flanking barcode sequences and initiates the amplification of the barcoded DNA fragment, while the higher concentration of Aoligos in the PCR mixture subsequently took over to amplify the barcoded DNA fragment further.^3^ The time required for CompetePCR is equivalent to that of the CLIVA protocol.^3^ This approach generated DNA fragments with phosphorothioate-modified nucleotides suitable for sticky end generation via an iodine-mediated reaction. This workflow has been successfully implemented for routine plasmid construction, with DNA fragments up to 6.5 kb being successfully cloned to date.

### Alternative plasmid assembly methods

Despite the significance of plasmids in biotechnology, existing plasmid construction methods are limited by inefficiencies in plasmid design and modification.^2^ Traditional cloning techniques, such as the restriction-enzyme-based method, rely on specific recognition sequences for restriction enzymes to create sticky ends (Figure 1A).^76^^,^^81^ Restriction enzymes cut DNA at specific recognition sequences, typically 4–8 base pairs in length, which limits flexibility if these sequences are absent at the desired location (Figure 1A).^82^ The limited availability of restriction enzymes, each with unique recognition sites, further complicates generating sticky ends at specific positions.^82^ While recognition sites can be introduced into DNA fragments to facilitate restriction enzyme use, this often leaves “scars” at the junction sites, which may interfere with downstream applications or alter plasmid functionality.^2^^,^^76^^,^^82^ Such modifications complicate customization and standardization, particularly when multiple compatible fragments are required.^83^ For multiple inserts, restriction-enzyme-based cloning becomes labor intensive, requiring repetitive cycles of digestion, ligation, and transformation, which is inefficient compared to modern methods like Gibson assembly that incorporate multiple fragments in a single step.^83^ Additionally, internal restriction sites within DNA fragments must be removed or avoided to prevent undesired cuts, further limiting plasmid design flexibility.^83^ In contrast, the CompetePCR workflow is not constrained by recognition sequence availability, as any sequence can be phosphorothioate modified via PCR, enabling the generation of 15-nt sticky ends through an iodine-mediated reaction (Figure 1D).^2^^,^^3^ Additionally, CompetePCR, like Gibson assembly, does not leave any scarring after plasmid assembly, resulting in seamless constructs.^2^^,^^3^

More advanced methods, such as Gibson assembly, enable the seamless joining of DNA fragments without the need for restriction sites (Figure 1B).^74^^,^^84^ Gibson assembly involves a mixture of three enzymes: exonuclease, polymerase, and ligase (Figure 1B).^74^^,^^84^ The exonuclease generates sticky ends by chewing back the 5′ ends of DNA fragments, facilitating the annealing of complementary regions, although the length of these sticky ends is not precisely controlled (Figure 1B).^74^^,^^84^ DNA polymerase subsequently fills in the gaps between the annealed fragments, and ligase seals the nicks to form a continuous DNA strand, resulting in a scarless construct (Figure 1B).^74^^,^^84^ Despite its advantages, Gibson assembly can be costly due to the requirement for multiple enzymes, making it less accessible for laboratories with limited budgets or those conducting high-throughput assemblies.^85^ In contrast, the CompetePCR workflow enables the controlled generation of a 15-nt sticky end without requiring the use of exonuclease (Figure 1D). Recombinant DNA ligase is also not required to ligate different DNA fragments together in an *in vitro* ligation reaction.^1^^,^^2^^,^^3^ Instead, the assembled fragments are ligated within the *E. coli* cell through an *in vivo* process, thereby reducing the overall cost of the procedure.^1^^,^^2^^,^^3^ Plasmid sequencing has confirmed that constructing plasmids using phosphorothioate-modified bonds yields results identical to other methods, such as restriction-enzyme-based cloning and Gibson assembly.^2^

Each plasmid assembly method has its merits and may be uniquely suited for various plasmid designs (Table S1). Restriction enzymes have been in use for over 50 years to generate sticky ends for plasmid assembly, and its use is established in the current synthetic biology field. It is highly suitable if the DNA fragment is very large (>50 kb) as PCR amplification may be challenging. It is critical that the recognition and cut site of restriction enzymes to be unique to cut only at the desired location. If the cut sites are incompatible with various DNA fragments and the DNA fragments are moderately large (∼20 kb to 50 kb), Gibson assembly may be considered. For plasmids less than 20 kb in length, the use of phosphorothioate-modified oligonucleotides may be suitable.

### Overview of gene design and availabilities of DNA materials

**Figure 2:**
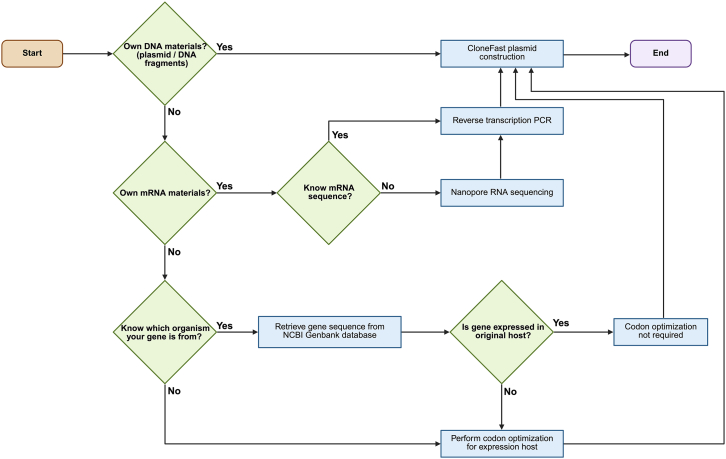
Decision flowchart for gene design and plasmid construction using the CloneFast workflow The flowchart outlines the steps to determine the appropriate procedure for plasmid construction based on the availability of DNA or mRNA materials, gene sequence information, and expression host requirements. Key steps include determining whether the mRNA sequence is known, retrieving gene sequences from the NCBI GenBank database, performing reverse transcription PCR, and determining if codon optimization is needed.

The gene design process begins by determining the availability of the necessary DNA materials, such as plasmids or DNA fragments (Figure 2). If these materials are available, proceed with the CloneFast plasmid construction workflow. If DNA materials are not available, the next step is to evaluate whether mRNA materials are accessible. If mRNA is available and the sequence is known, reverse transcription PCR can be performed to convert mRNA into complimentary DNA (cDNA), followed by CloneFast plasmid construction. If the mRNA sequence is unknown, Nanopore RNA sequencing can be conducted to determine the sequence before proceeding with reverse transcription and subsequent plasmid construction. If neither DNA nor mRNA materials are available, it is necessary to determine whether the source organism of the gene is known. If the organism is known, the gene sequence can be retrieved from the National Center for Biotechnology Information (NCBI) GenBank, which is a public database. After retrieving the gene sequence, it is important to assess whether the gene is expressed in its original host. If it is expressed in the original host, codon optimization is not required, and the gene can be used directly.^86^^,^^87^^,^^88^^,^^89^^,^^90^ However, if the gene is not expressed in the original host, codon optimization must be performed to adapt the sequence for the intended expression host.^91^^,^^92^^,^^93^^,^^94^ Once codon optimization is complete, CloneFast plasmid construction can proceed.

### Overview of plasmid design and plasmid construction workflow

**Figure 3:**
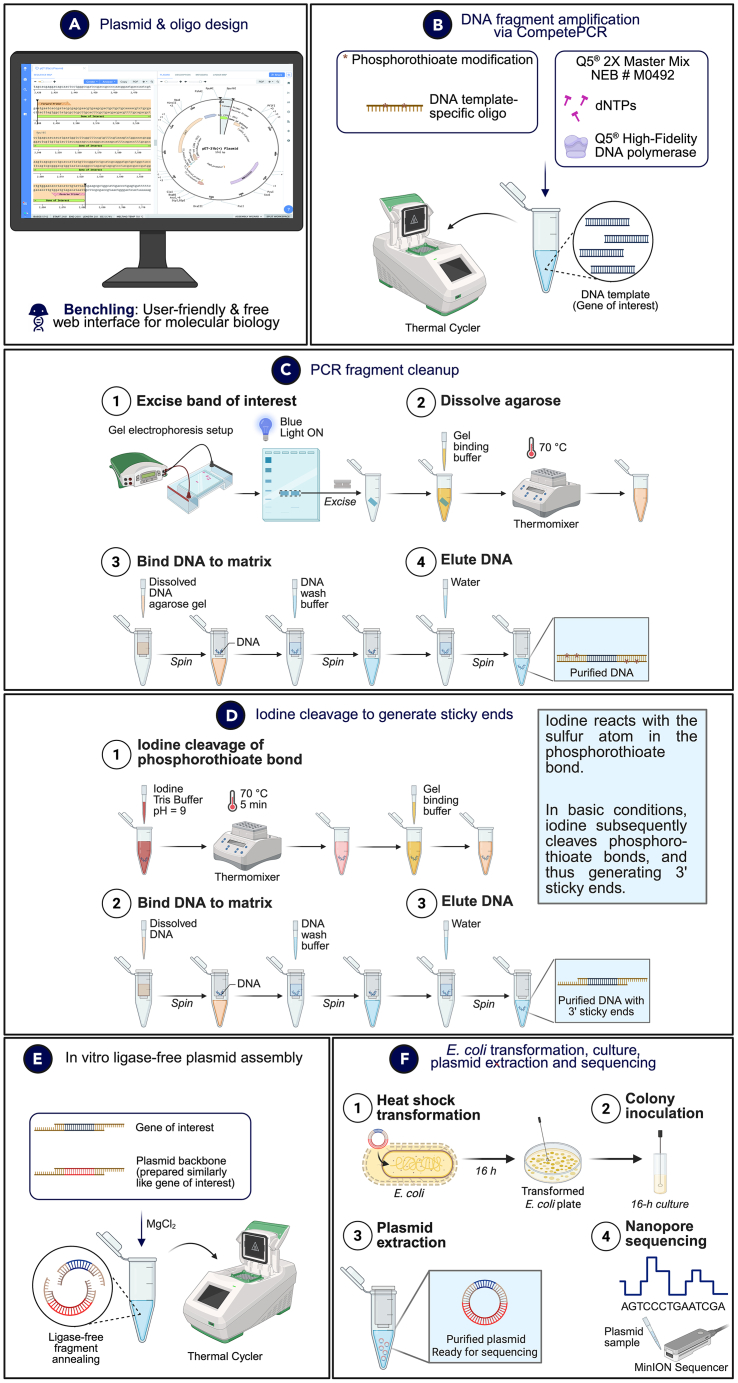
Overview of CloneFast workflow for plasmid construction (A) Design new plasmids and oligos for plasmid construction using Benchling, a user-friendly web interface for molecular biology. (B) Amplify DNA template fragments with Q5 High-Fidelity 2X Master Mix using oligos containing phosphorothioate-modified bonds in a thermal cycler. (C) Perform standard PCR fragment cleanup, including horizontal gel electrophoresis to resolve DNA bands, followed by excision of the correct band and DNA gel extraction and purification. (D) Cleave phosphorothioate-modified bonds in the PCR product through a 5-min iodine-mediated reaction at pH 9 and 70°C to generate 3′ sticky ends, followed by DNA fragment purification. (E) Conduct an *in vitro* ligase-free plasmid assembly in a thermal cycler by combining the gene of interest and plasmid backbone fragments containing 3′ sticky ends. (F) Perform heat shock transformation to introduce the assembled plasmid into *E. coli*, inoculate a single colony into liquid culture for 16-h growth, and extract and purify the plasmid for Nanopore sequencing to verify the sequence.

Prior to plasmid construction, researchers would first perform fundamental bioinformatics techniques to design plasmids and oligos *in silico* using Benchling, a user-friendly and freely accessible web interface for molecular biology (Figure 3A). Subsequently, researchers would execute the CompetePCR workflow for amplifying DNA fragments using the oligos that were designed (Figure 3B). Researchers are encouraged to refer to this journal article for detailed instructions in executing the CompetePCR workflow.^3^ Standard DNA column purification techniques for isolating DNA fragments can be used (Figure 3C). Researchers would then perform the 5-min iodine-mediated reaction for generating sticky ends by cleaving phosphorothioate bonds (Figure 3D). Researchers are encouraged to refer to this journal article for detailed instructions and background in the use of iodine to cleave phosphorothioate bonds to generate sticky ends.^1^^,^^2^^,^^3^ Iodine cleaves phosphorothioate bonds via oxidative desulfurization, where iodine acts as an oxidant that reacts with the sulphur atom in the phosphorothioate bond, and hydrolysis occurs at basic conditions, which facilitates the cleavage of the phosphorothioate bond.^95^ It is critical to adhere to the 5-min iodine treatment timing to generate sticky ends efficiently. The 5-min iodine cleavage reaction is highly efficient (∼100%), and the reaction is optimal at pH 9 in Tris buffer (final concentration: 10 mM).^95^ Subsequently, researchers can perform an *in vitro* ligase-free plasmid assembly using magnesium chloride (Figure 3E). Lastly, researchers would perform the heat shock transformation procedure for introducing the plasmid into the cloning host *Escherichia coli*, subsequent *E. coli* culture, plasmid extraction, plasmid purification, and Nanopore sequencing to verify plasmid sequence integrity (Figure 3F). A detailed protocol can be referred from the supplemental information.

### *In silico* native gene sequence and information retrieval

**Figure 4:**
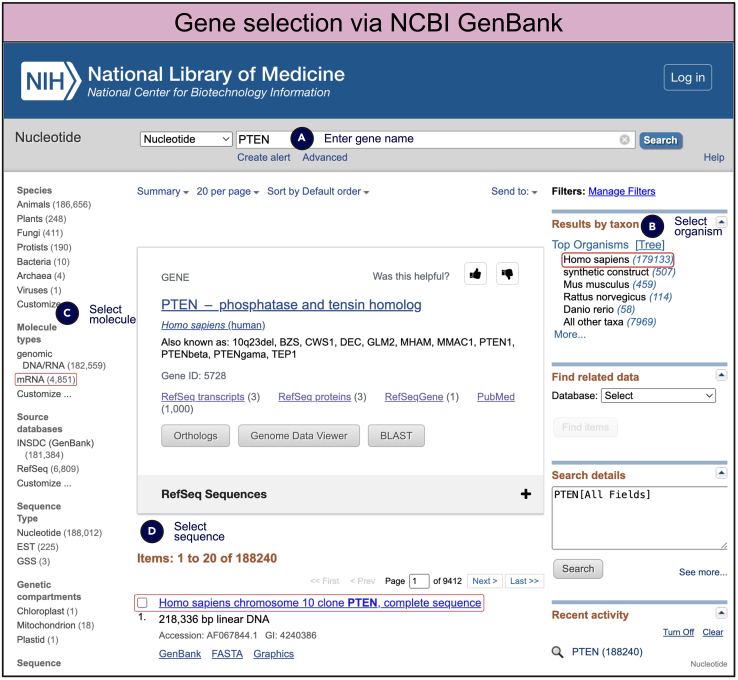
Gene selection and retrieval from NCBI GenBank for plasmid construction (A) The gene of interest, such as phosphatase and tensin homolog (PTEN), is searched using the GenBank interface. (B) The organism can be specified by selecting from the “results by taxon” section to narrow down the search, ensuring that the selected sequence is relevant to the target organism. (C) Molecular type, such as mRNA or DNA, can be refined under “molecular types” to obtain the desired genetic material. If coding sequence is of interest, select “mRNA.” (D) Once the appropriate sequence is located, it can be selected from the results for subsequent use in plasmid construction.

If existing DNA or mRNA materials are not available, gene sequences can be retrieved from the NCBI GenBank (https://www.ncbi.nlm.nih.gov/genbank/) by searching for the gene of interest. Phosphatase and tensin homolog (PTEN) gene is used here as an example (Figure 4A). To refine your search, specify the desired organism (e.g., Homo sapiens or Mus musculus, commonly used for biomedical research) by selecting the organism in the “results by taxon” section (Figure 4B). If the goal is to express a protein in a mammalian host cell, refine the search by selecting the appropriate molecular type (e.g., mRNA) under “molecular types” (Figure 4C). For eukaryotic coding sequences, alternative splicing may result in multiple transcript variants for the same gene. If a specific transcript variant is required, it can be selected from the refined results in NCBI GenBank (Figure 4D).

### Guidelines on choosing gene variants and codon optimization

**Figure 5:**
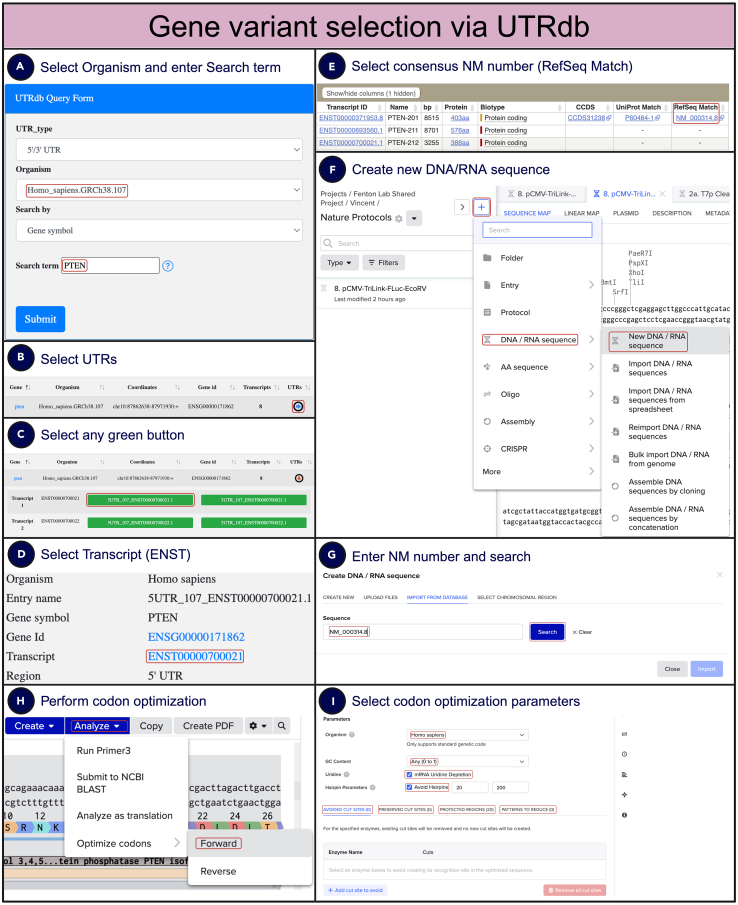
Selection of gene variants via UTRdb and codon optimization via Benchling (A) The UTRdb query form is used to search for a gene of interest by selecting the organism and entering the gene name. (B) From the table of results, users can select the appropriate UTR sequence for further analysis. (C) Green buttons indicate available transcript variants, and clicking on one provides detailed information about each variant. (D) The selected transcript (ENST) is displayed, providing essential information such as gene ID and organism. (E) The canonical RefSeq match number, starting with “NM,” is selected to identify the most common transcript variant. (F and G) In Benchling, a new DNA/RNA sequence is created (F), and the RefSeq match number is used to import the desired sequence (G). (H) The imported sequence is annotated, making it easy to identify coding regions. (I) For codon optimization, the “analyze” option is used to optimize codons, allowing users to specify parameters such as GC content, uridine content, and regions to avoid or protect during optimization. Additional codon optimization parameters can be set to reduce specific DNA sequence patterns or preserve specific enzyme cut sites. This figure provides a streamlined guide to selecting and optimizing gene sequences for expression, ensuring compatibility with the intended host organism.

If the required transcript variant is unknown, it is recommended to use the most common, canonical variant. To identify this variant, use the UTRdb database (https://utrdb.cloud.ba.infn.it/utrdb/search107.html). Customize the UTRdb query form by selecting the organism and entering the gene name (Figure 5A). A table of results will be generated (Figure 5B). Click the blue arrow in the rightmost cell under the “UTRs” header to display 5UTRs and 3UTRs (Figure 5B). Click on any green button (Figure 5C), and a new webpage with a transcript number will be generated (Figure 5D). Click on the blue text starting with “ENST” under the “transcript” column (Figure 5D). The canonical sequence can be identified under the “RefSeq match” header, indicated by the “NM” prefix (Figure 5E). Copy the RefSeq match number. Next, open Benchling, create a new DNA/RNA sequence, and use the “import from database” option (Figure 5F). Paste the RefSeq match number in the sequence search bar and press “search” to generate the sequence (Figure 5G). The sequence will be annotated, and the coding sequence can be easily identified. If codon optimization is required, click “analyze,” and then “optimize codons” (Figure 5H). Specify the organism for optimization, GC content, uridine content, and whether to avoid hairpins (Figure 5I). Use the “avoided cut sites” option to remove existing cut sites and prevent the formation of new ones for the specified enzymes. The “preserved cut sites” option protects existing sites from modification. Regions that should not be altered during optimization can be designated as “protected regions,” and specific DNA sequence patterns to reduce can be defined using the “patterns to reduce” option.

### Plasmid and oligo design

**Figure 6:**
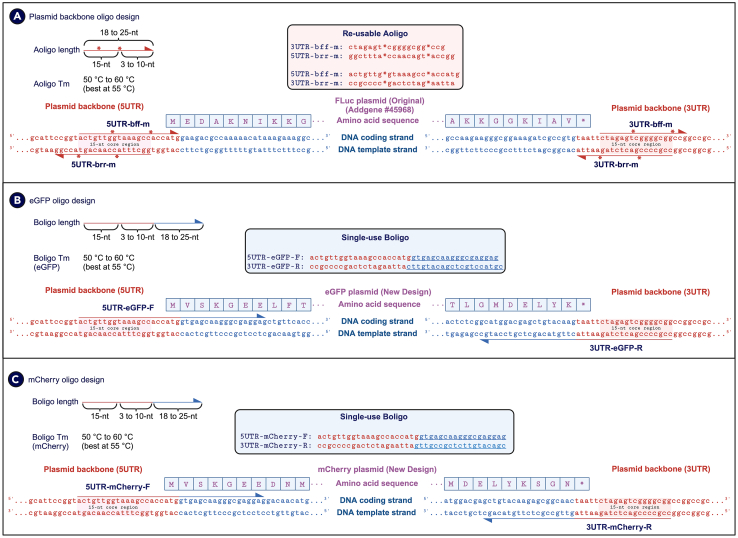
Design of Aoligos and Boligos for CloneFast plasmid construction (A) A plasmid (Addgene #45968) encoding FLuc was used to demonstrate the design of Aoligos for DNA fragment amplification using CompetePCR. FLuc DNA sequences are shown in blue, while the 5′ and 3′ untranslated regions (5UTR and 3UTR) are shown in red. The amino acid sequences are shown in purple. The direction of the Aoligo and Boligo sequence is depicted via the half arrow heads (e.g., left to right). The Aoligos are designed to span the plasmid backbone regions with lengths between 18 and 25 nt, incorporating two phosphorothioate modifications positioned within the 15-nt core region. The Tm of Aoligos is optimized to be between 50°C and 60°C, with an optimal Tm of approximately 55°C, ensuring reliable amplification. (B) To replace the FLuc gene with eGFP, Boligos are designed with two distinct regions. The outer region is identical to the Aoligo but lacks phosphorothioate modifications, while the inner region is specific to the gene of interest and ranges between 18 and 25 nt in length. (C) To replace FLuc gene with mCherry, Boligos for mCherry are designed similarly with eGFP, where only the inner region corresponding to the gene-specific sequence in blue are changed. Since the outer region sequence corresponding to the plasmid backbone sequence flanking the eGFP or mCherry remains constant, it demonstrates that any gene of interest can be barcoded using the same Aoligo sequences. The ability to reuse Aoligos significantly reduces the cost of plasmid construction.

To design oligos for plasmid construction, the following principles should be adhered to, as illustrated in the provided guide (Figure 6). It is strongly recommended to use the Benchling web interface for visualizing plasmid and oligo designs. In this example, a plasmid (Addgene #45968) encoding firefly luciferase (FLuc) is used to design Aoligos that generate DNA fragments compatible with CompetePCR (Figure 6A). The FLuc DNA sequences are indicated in blue text, while the left-flanking 5′ untranslated region (5UTR) and right-flanking 3′ untranslated region (3UTR) sequences on the plasmid backbone are indicated in red text (Figure 6A). The top DNA coding strand is read from the 5′ to 3′ direction (left to right), while the bottom DNA template strand is read from the 5′ to 3′ direction (right to left) (Figure 6A). The amino acid sequence of FLuc is indicated in purple text (Figure 6A). Since most protein-coding genes begin with a start codon (ATG) and terminate with a stop codon (typically TAA, although TGA and TAG are also possible), these start and stop codons can be considered part of the plasmid backbone (Figure 6A).

The first step involves determining the length of the Aoligos. The reusable Aoligos (indicated in red text) are designed to recognize the plasmid backbone sequence region (also indicated in red text) (Figure 6A). The Aoligo should have a total length of 18 to 25 nt, incorporating two phosphorothioate modifications (denoted by ∗) strategically positioned within the 15-nt core region, with an additional 3 to 10 nt beyond the second phosphorothioate-modified bond (Figure 6A). The total length of the Aoligo should be optimized by adjusting the sequence to achieve a melting temperature (Tm) between 50°C and 60°C, with an optimal Tm of approximately 55°C (Figure 6A). Tm deviations from 55°C may pose some challenge for the primer to anneal to the DNA template. The melting temperature can be determined in Benchling automatically when the sequence of choice is highlighted.

To initiate the design of Aoligos, begin by designing the pair of Aoligos (5UTR-bff-m and 3UTR-brr-m) that will flank the gene of interest, adhering to the guidelines provided for Tm and the 15-nt core region. This step should be followed by designing the remaining pair of Aoligos (5UTR-brr-m and 3UTR-bff-m) (Figure 6A). To verify the accuracy of the Aoligo design, visually inspect the following criteria. For the left side of the plasmid backbone (5UTR), ensure that the inner phosphorothioate modification on the 5UTR-brr-m Aoligo coincides with the beginning of the 5UTR-bff-m Aoligo and that the inner phosphorothioate modification on the 5UTR-bff-m Aoligo coincides with the beginning of the 5UTR-brr-m Aoligo (Figure 6A). The distance between the inner phosphorothioate modifications of both the 5UTR-brr-m and 5UTR-bff-m Aoligos should be 15 nt (Figure 6A). Similarly, for the right side of the plasmid backbone (3UTR), ensure that the inner phosphorothioate modification on the 3UTR-brr-m Aoligo coincides with the beginning of the 3UTR-bff-m Aoligo and that the inner phosphorothioate modification on the 3UTR-bff-m Aoligo coincides with the beginning of the 3UTR-brr-m Aoligo (Figure 6A). The distance between the inner phosphorothioate modifications of both the 3UTR-brr-m and 3UTR-bff-m Aoligos should also be 15 nt (Figure 6A).

To replace the FLuc gene sequence with other genes of interest, such as eGFP or mCherry, while maintaining the flanking plasmid backbone 5UTR and 3UTR sequences (Figures 6B and 5C), a pair of unmodified Boligos must be designed. Each Boligo comprises two distinct regions: an outer region (indicated in red text), which is identical to the Aoligo sequence but lacks phosphorothioate modifications, and an inner region (indicated in underlined blue text), which is specific to the outer complementary ends of the gene of interest (Figures 6B and 6C). The length of the inner region specific to the gene of interest should range from 18 to 25 nt. The Tm of the inner region should be between 50°C and 60°C, with an optimal Tm of approximately 55°C (Figures 6B and 6C). It is important to note that for Boligos in both eGFP and mCherry examples, the outer regions (indicated in red text) are identical, while the inner regions (indicated in underlined blue text) differ depending on the gene of interest (Figures 6B and 6C). This demonstrates that any gene of interest can be barcoded using the same pair of Aoligo sequences. Refer to Figure S1 and the key resources table in the supplemental information for the oligo sequence used.

### Required research equipment

Most reagents used in synthetic biology require cold storage, and this protocol is no exception. Specifically, this protocol requires storage at 4°C for bacterial agar plates following plasmid transformation, −20°C for Q5 High-Fidelity 2X Master Mix, oligos used for PCR and antibiotic aliquots, and −80°C for *E. coli* competent cells and glycerol stocks of *E. coli* strains containing verified plasmid constructs. The freezer costs approximately USD $200.

A thermal cycler is required for DNA fragment amplification and assembly. Additionally, depending on the scale of plasmid construction and the number of researchers involved, acquiring multiple thermal cyclers may be beneficial to enhance process throughput.

A gel documentation system is a laboratory instrument used to visualize and document nucleic acids, such as DNA and RNA, as well as protein samples resolved by gel electrophoresis. However, for plasmid construction, only the visualization of DNA is required. We recommend using a blue light transilluminator (∼USD $130) that allows visualization of nucleic acids, enabling users to visualize and excise the correct DNA band for subsequent purification. It is important to avoid using an UV transilluminator for this application, as UV light can be harmful to the eyes and skin. Blue light is a safer alternative.

A dedicated cell incubator for *E. coli* agar plate incubation is required to obtain bacterial colonies. It is crucial to avoid sharing the incubator with other types of cell cultures, such as mammalian cell cultures, to prevent cross-contamination. Typically, *E. coli* agar plates are incubated at 37°C for 16 h; however, in certain cases, particularly when plasmid constructs involve highly repetitive sequences (e.g., polyA sequence), an incubation temperature of 30°C or lower is recommended to minimize homologous recombination.^96^ If a dedicated incubator is not available, agar plates can be left on the laboratory bench at 25°C for a 2-day incubation. For culturing single colonies in liquid culture, an orbital shaker is required to agitate the *E. coli* culture, as agitation promotes cell growth and provides the necessary aeration.

A biological safety cabinet is crucial for maintaining sterility during the handling of *E. coli* transformants, including spreading cells on agar plates and inoculating single colonies in liquid culture, thereby minimizing contamination. Contrary to common misconceptions, the antibiotics used in agar plates and liquid cultures are not intended to prevent contamination.^97^^,^^98^ Many laboratory contaminants can overcome commonly used antibiotics.^99^^,^^100^ The primary purpose of using antibiotics is to create selective pressure, ensuring that *E. coli* maintains the plasmid to achieve high plasmid yield and titer.^3^^,^^101^ Therefore, the use of a biological safety cabinet is necessary when handling live *E. coli* to minimize contamination. Usage of 70% ethanol for regular disinfection is thus recommended.

## Reagent preparation and equipment calibration

### Biosafety considerations

Biosafety requirements may vary depending on the plasmid type and host organism. For example, the non-pathogenic *E. coli* DH5α used in this primer is commonly used for generic plasmid construction. It is genetically modified and lacks virulence factors, hence it poses minimal risk under biosafety level 1 (BSL-1) conditions. The constructed plasmid can then be introduced to the host organism of choice. If pathogenic host strains are used, relevant regulatory guidelines should be consulted to determine the appropriate containment and biosafety levels.

### Best practices for handling biological materials and reagents

When handling DNA, it is crucial to exercise great care due to its fragile nature. DNA is highly susceptible to shearing by physical stress, making it prone to damage during routine laboratory procedures.^102^^,^^103^ Vigorous mixing or pipetting must be strictly avoided, as these actions introduce shearing forces that can break DNA strands.^102^^,^^103^ Instead, gentle pipetting or careful mixing, such as tube flicking, is recommended to maintain DNA integrity.^102^^,^^103^ When resuspending DNA, slow and controlled motions should be used to ensure proper handling.^102^^,^^103^ The delicate nature of DNA requires meticulous attention to handling techniques to avoid compromising experimental results and to ensure high-quality preparations for downstream applications, such as PCR and plasmid extraction.^102^^,^^103^ Proper care during DNA handling not only preserves the integrity of the sample but also significantly contributes to the success of molecular biology experiments.^102^^,^^103^

When handling reagents, strict adherence to best practices is crucial to minimize contamination. All procedures involving culturing *E. coli* should be performed within a biological safety cabinet to maintain sterility. Ensure that all laboratory consumables, including pipettes, pipette tips, and serological pipettes, are sterile and DNase/RNase-free prior to use. Pre-calculate the required reagent volumes for the experiment and aliquot the necessary amounts from master stocks to minimize contamination risk and reduce reagent wastage. It is essential not to return unused reagents to the master stock, as this could introduce contaminants.

### Reconstitution of oligos

Upon receipt of the oligos, centrifuge the tube for 1 min at 25°C to ensure that the contents are collected at the bottom. Identify the oligo yield in nmol, which can be found on the tube label or the specification sheet provided by Azenta. Multiply this value by 10 to determine the volume of IDTE buffer (in μl) required to reconstitute the oligo to a final stock concentration of 100 μM. For instance, for an oligo yield of 9 nmol, add 90 μl of IDTE buffer. Incubate the reconstituted oligo at 25°C in a thermomixer at 700 rpm for 30 min. Subsequently, spin down the tube, at 20,000 × *g* for 1 min at 25°C, before storing at −20°C. The reconstituted oligos are stable at this temperature for several years.

### Agarose-TAE gel (0.8% w/v) with SYBR Safe DNA Gel Stain

Prepare 400 mL of 1X TAE buffer by dissolving 8 mL of 50X TAE buffer with 392 mL of deionized water at 25°C. Set aside 300 mL of 1X TAE buffer to fully submerge the solidified agarose-TAE gel. This ensures optimal electrical conductivity during electrophoresis. Combine 0.8 g of agarose with 100 mL of 1X TAE buffer in a 500-mL duran bottle and microwave until fully dissolved. Add 10 μl of 10,000X SYBR Safe DNA Gel Stain to the molten agarose. Swirl and mix well and pour into an appropriate gel-casting tray with an 8-well comb. Leave the gel at 25°C to solidify for 30 min or until the gel is nearly opaque. The solidified gel should be used within the same day. Load the gel onto the horizontal gel electrophoresis chamber.

### Iodine solution (stock concentration: 30 g/L^−1^)

Combine 30 mg of iodine with 1 mL of molecular biology grade pure ethanol at 25°C to make a 30 g/L^−1^ iodine solution. Vortex the solution thoroughly to ensure proper mixing. Store the solution in a 2-mL microcentrifuge tube, protected from light, at 25°C. The solution remains stable under these conditions for several years.

### Magnesium chloride solution (stock concentration: 10 mM)

Combine 9.5 mg of magnesium chloride with 10 mL of molecular biology grade water at 25°C to make a 10-mM magnesium chloride solution. Sterilize the magnesium chloride solution by passing through a 0.22-μm PES filter. Aliquot the sterile solution into 2-mL microcentrifuge tubes. The solution remains stable for several years at 25°C.

### Ampicillin solution (stock concentration: 100 mg mL^−1^)

Weigh out 500 mg of ampicillin sodium salt in a new 15-mL corning tube. Add 5 mL of molecular biology grade water and vortex the solution thoroughly until ampicillin is completely dissolved. Pass the ampicillin solution through a 0.22-μm PES filter for sterilization purposes. Avoid multiple freeze-thaw cycles by aliquoting the sterile solution into 1.7-mL microcentrifuge tubes (0.5 mL per tube). The ampicillin aliquots may be stored for at least a year at −20°C in the dark.

### Terrific Broth containing ampicillin

Dispense 10 mL of Terrific Broth into a 50-mL conical tube within a biological safety cabinet. Add 10 μl of ampicillin stock solution (100 mg mL^−1^) to the Terrific Broth. Prepare the ampicillin-containing Terrific Broth just before inoculating single *E. coli* colonies.

**CAUTION:** As antibiotics degrade over time, it is essential to freshly prepare the Terrific Broth containing antibiotics.

### Glycerol solution (60% v/v)

Make 60% (v/v) glycerol solution by diluting 100% glycerol in molecular biology grade water in the biological safety cabinet. Pass the glycerol solution through a 0.22-μm PES filter for sterilization purposes. The solution remains stable for several years at 4°C.

### Nanodrop spectrophotometer

The Nanodrop spectrophotometer is commonly used to measure nucleic acid concentration and quantify the purity of the nucleic acid sample. It is recommended to perform calibration at least every 6 months to verify if the Nanodrop equipment is operating within manufacturing specifications. The calibration protocol can vary depending on the model and brand of the Nanodrop equipment.

### Micropipette

The micropipette is a common equipment to accurately aliquot small liquid volumes. It is recommended to engage an external vendor to perform calibration at least every year.

## Limitations

The use of phosphorothioate-modified oligonucleotides to construct plasmids has been successfully demonstrated for up to seven DNA fragments (Table S2). Gibson assembly may be considered to assemble more than seven fragments. The use of PCR to amplify DNA fragments may present a limitation in workflows such as CompetePCR, as well as in other plasmid construction methodologies, including restriction enzyme cloning and Gibson assembly. It is commonly perceived that DNA polymerases, including high-fidelity variants such as Q5 High-Fidelity DNA Polymerase, may introduce unwanted mutations during PCR amplification.^104^^,^^105^ However, this concern can be effectively mitigated through whole plasmid sequencing via Nanopore sequencing, ensuring the accuracy and integrity of the final plasmid construct. Therefore, we do not anticipate this to be a significant issue.

The use of phosphorothioate-modified oligonucleotides has assembled DNA fragment length up to 6.5 kb. Researchers using PCR to routinely amplify DNA fragments much larger than 6.5 kb may consider specialized ultra-long high-fidelity DNA polymerases such as LA Taq DNA polymerase from Takara Bio (up to 48 kb), which are subsequently assembled using Gibson assembly.

Phosphorothioate-modified oligonucleotides have been used to construct large plasmids (e.g., 21.6 kb in total length) for metabolic engineering and synthetic biology purposes. These plasmids are designed for general protein expression in common hosts such as *E. coli*, yeast, and mammalian cells. The insertion of short guide RNA sequence (e.g., 36 bp) in CRISPR-Cas9 plasmids for gene editing was also demonstrated. Gibson assembly may also be used to assemble for much larger plasmids.

## Conclusion

The CloneFast plasmid construction guide addresses current challenges faced by labs in constructing plasmids economically and efficiently. By harnessing phosphorothioate-modified oligonucleotides, this methodology simplifies the generation of precise sticky ends, overcoming the limitations commonly associated with traditional cloning techniques (restriction enzyme and Gibson assembly), such as restriction enzyme cut site dependence, introducing sequence scarring in plasmid design, and the associated high costs of enzymes. CloneFast allows scarless assembly of DNA fragments, providing significant cost savings through reusable oligonucleotides and *in vitro* ligase-free procedures. This approach has demonstrated success, suggesting broad applicability in metabolic engineering, synthetic biology, and gene therapy. Importantly, the ease of *in silico* plasmid design using online platforms such as Benchling ensures accessibility to diverse research communities, enabling rapid customization and iterative plasmid modifications. As molecular cloning techniques continue to advance, workflows like CloneFast are poised to become integral, particularly for laboratories aiming for flexibility, cost-effectiveness, and expedited plasmid construction to address emerging biotechnological challenges.

## Acknowledgments

The graphical abstract and Figures 1, 2, 3, 4, 5, 6, S1, and S2 were created by the authors as original figures on BioRender.com. We thank Tara J. Skelly in the High-Throughput Sequencing Facility for their assistance in Nanopore RNA sequencing. This facility is supported by the University Cancer Research Fund, Comprehensive Cancer Center Core Support Grant (P30-CA016086), and UNC Center for Mental Health and Susceptibility Grant (P30-ES010126). This work was supported by an 10.13039/100000052NIH National Institute of Biomedical Imaging and Bioengineering award (1R21EB034942-01). This work was also supported by the NC Translational and Clinical Sciences (10.13039/100011485NC TraCS) Institute, which is supported by the NIH National Center for Advancing Translational Sciences (10.13039/100006108NCATS) award 1K12TR004416-01. We extend our gratitude to Sam Lai for generously providing access to his biological safety cabinet and Nanodrop spectrophotometer. Lastly, we would like to acknowledge Kang Zhou’s research laboratory from the National University of Singapore for the development of CompetePCR workflow, which uses phosphorothioate-modified oligonucleotides for routine plasmid construction.

## Author contributions

V.F.: conceptualization, methodology, investigation, formal analysis, and writing – original draft. P.B.T.: conceptualization, methodology, investigation, formal analysis, and writing – original draft. O.S.F.: supervision, writing – review & editing, and funding acquisition.

## Declaration of interests

The authors declare no competing interests.

## Supplemental information

Document S1. Supplemental protocol including key resources table, step-by-step method details, troubleshooting, Figures S1–S3, and Tables S1 and S2
