# Supplementary material for: CloneFast: A simple plasmid design and construction guide for labs venturing into synthetic biology
Source: STAR Protoc. 2025 Aug 6;6(3):104025. doi: 10.1016/j.xpro.2025.104025 (PMC12355518; doi:10.1016/j.xpro.2025.104025)
Supplement: Document S1. Supplemental protocol including key resources table, step-by-step method details, troubleshooting, Figures S1–S3, and Tables S1 and S2 [file mmc1.pdf]

Supplementary Information for

CloneFast: A simple plasmid design and construction guide for labs venturing into synthetic biology

Vincent Fung,[1,2] Palas Balakdas Tiwade,[1,2] and Owen S. Fenton[1,*]

[1]Division of Pharmacoengineering and Molecular Pharmaceutics, Eshelman School of Pharmacy, University of North Carolina at Chapel Hill, Chapel Hill, NC 27599, USA

[2]Vincent Fung and Palas Balakdas Tiwade have contributed equally to the protocol development.

*Correspondence: osfenton@unc.edu

Key resources table

REAGENT or RESOURCE	SOURCE	IDENTIFIER
Bacterial and virus strains		
NEB 5-Alpha Competent E. coli (High Efficiency)	NEB	Cat# C2987H
Chemicals, peptides, and recombinant proteins		
Water, Molecular Biology Grade	Corning	Cat# 46-000-CM
70% ethanol	VWR	Cat# BDH1164-4LP
Ethanol, Absolute (200 Proof), Molecular Biology Grade	Fisher Scientific	Cat# BP28184
IDTE Solution (10 mM Tris, 0.1 mM EDTA), pH 8.0	IDT	Cat# 11-01-02-05
Tris 1.0 M Solution, pH 9.0	VWR	Cat# 97062-940
Iodine	Sigma-Aldrich	Cat# 207772
Magnesium Chloride, Anhydrous	Sigma-Aldrich	Cat# M8266-100G
50X Tris-Acetate-EDTA, (TAE) Buffer	Fisher Scientific	Cat# BP1332-1
Agarose	Research Products International	Cat# A20090-100.0
Ampicillin Sodium Salt	VWR	Cat# 71003-302
Glycerol	Genesee Scientific	Cat# 18-205
10,000X SYBR Safe DNA Gel Stain	Invitrogen	Cat# S33102
Gel Loading Dye, Purple (6X)	NEB	Cat# B7024S
Quick-Load Purple 1 kb Plus DNA Ladder	NEB	Cat# N0550S
Terrific Broth	Thermo Fisher Scientific	Cat# A1374301
SOC Outgrowth Medium	NEB	Cat# B9020SVIAL
Critical commercial assays		
ZymoPURE Plasmid Miniprep Kit	Zymo Research	Cat# D4210
One*Taq* RT-PCR Kit	NEB	Cat# E5310S
GeneJET Gel Extraction Kit	Thermo Fisher Scientific	Cat# K0692
Q5 High-Fidelity 2X Master Mix	NEB	Cat# M0492S
Oligonucleotides		
Aoligos:<br>TriLink FLuc cDNA sequence:<br>P_{T7}-TriLink FLuc-bff-m: atacgac*tcactata*agg<br>SV40 polyA-TriLink FLuc-brr-m: caatgta*tcttagat*atc<br><br>Plasmid backbone:<br>SV40 polyA-bff-m: atctaaga*tacattg*atgagtttgg<br>P_{T7}-P_{cmv}-brr-m: tatagtga*gtcgtat*taagctctgc	Azenta Life Sciences	
Boligos:<br>TriLink FLuc cDNA sequence:<br>P_{T7}-TriLink FLuc-F:<br>atacgactcactataaggaaataagagagaaaagaagagt<br>SV40 polyA-TriLink FLuc-R:<br>caatgtatcttagatatcttcctactcaggctttattcaa<br><br>Plasmid backbone:<br>SV40 polyA-F: atctaagatacattgatgagtttggaca<br>P_{T7}-P_{cmv}-R: tatagtgagtcgtattaagctctgcttatatagacctccc	Azenta Life Sciences	
Recombinant DNA		
Plasmid (FLuc, mammalian expression)	Addgene	Cat# 45968
CleanCap FLuc mRNA (5moU)	TriLink Biotechnologies	Cat# L-7202

Software and algorithms		
MATLAB	MathWorks	Version: 2022a
Other		
Ice Maker Machine	Hoshizaki	F-500BAJ
C1000 Touch Thermal Cycler	Bio-Rad	C1000
Microwave	Panasonic	NN-H665WF
PowerPac Basic Power Supply for Electrophoresis	Bio-Rad	PowerPac 200
Horizontal Electrophoresis System, 7 x 10 cm	VWR	89032-290
Blue Light Transmitter	Fisher Scientific	S99499
Scalpel or penknife blade	Generic	N/A
Eppendorf Thermomixer C	Eppendorf	5382
Class II Biological Safety Cabinet	The Baker Company	SterilGARD III Advance SG403
Heratherm Cell Incubator	Thermo Fisher Scientific	S37377
Orbital Shaker	New Brunswick Scientific	EXCELLA E24R
Microcentrifuge	Eppendorf	5424
NanoDrop One	Thermo Fisher Scientific	ND-ONE-W
Dual 4 ˚C / -20 ˚C Freezer	EUHOMY	RF-H/RF-S
-80 ˚C freezer	PHCbi	MDF-DU702VH-PA
LB Agar Plates containing Ampicillin (100 μg ml^{-1})	UNC Tissue Culture Facility or VWR	AGP or Cat# 77443-376
Duran Bottle, 500-ml	Duran	Cat# 218014459
Microcentrifuge Tube, 1.7-ml	Genesee Scientific	Cat# 24-282S
Microcentrifuge Tube, 2.0-ml	Genesee Scientific	Cat# 24-283S
Conical Centrifuge Tube, 50-ml	Genesee Scientific	Cat# 21-106
Conical Centrifuge Tube, 15-ml	Genesee Scientific	Cat# 28-103
Syringe, 60-ml	VWR	Cat# 76124-668
Polyethersulfone (PES) 0.22-μm Filter	Millipore Sigma	Cat# SLGPB5010
PCR Tubes, 8-Strip, 0.2-ml, Sterile, DNase/RNase Free	Genesee Scientific	Cat# 27-125
Barrier Tip, 10-μl, Sterile, DNase/RNase Free	Genesee Scientific	Cat# 24-401
Barrier Tip, 20-μl, Sterile, DNase/RNase Free	Genesee Scientific	Cat# 24-404
Barrier Tip, 200-μl, Sterile, DNase/RNase Free	Genesee Scientific	Cat# 24-412
Barrier Tip, 1000-μl, Sterile, DNase/RNase Free	Genesee Scientific	Cat# 24-430
Sterile Serological Pipette, 50-ml	Genesee Scientific	Cat# 12-107ALP
L-Shape Spreader, Single pack	VWR	Cat# 76208-438

Step-by-step method details

RNA sequencing (optional)

● TIMING **~2 days (in-house) to 1 month (outsourced)**

1. RNA sequencing is only required if the starting material (gene-of-interest) is in the form of mRNA. Proceed to the next section if you have DNA starting materials such as plasmid or gBlocks for your gene-of-interest.

2. The coding sequence of CleanCap FLuc mRNA (5moU) is available online from TriLink Biotechnologies, though the 5UTR and 3UTR sequences flanking the mRNA are not disclosed. However, the total length of the CleanCap FLuc mRNA (5moU) is provided. We aimed to construct a plasmid encoding this specific mRNA for downstream applications such as UTR engineering. To determine the complete mRNA sequence, and to enable cloning into a plasmid, we performed RNA sequencing using Nanopore sequencing.

3. CleanCap FLuc mRNA (5moU) was sent to the UNC High-Throughput Sequencing Facility for RNA sequencing. The RNA was prepared using the cDNA-PCR Barcoding Kit V14 (SQK-PCB114.24). Sequencing was performed on a GridION system with a Flongle adapter (FLO-FLG114, pore version R10.4.1). A high accuracy basecalling algorithm was employed to generate the sequencing reads.

4. An in-house MATLAB algorithm was used to process the sequencing data. All sequencing reads were first filtered to retain those within 100 nucleotides of the full-length FLuc mRNA sequence (1922 nt). Subsequently, reads that passed the initial filter were aligned to the expected FLuc mRNA coding sequence to assess sequence matches and mismatches. The algorithm uses the Smith-Waterman algorithm to retain only reads with >95% sequence identity based on alignment. Sequencing reads that passed the second round were then multi-aligned to generate a consensus mRNA sequence. This process enabled elucidation of the 5UTR and 3UTR sequences, thereby facilitating the design of Boligos specifically recognizing the terminal regions of the 5UTR and 3UTR sequences. This process also enabled the design of a reverse primer for reverse-transcription PCR, which was used to generate cDNA, followed by cloning of the resulting DNA fragment into a plasmid.

In silico Aoligo and Boligo design for plasmid backbone and gene-of-interest

● TIMING **~10 min**

5. Addgene #45968 plasmid is designed for the mammalian expression of FLuc. However, the coding sequence, as well as the 5UTR and 3UTR, differ substantially from the CleanCap FLuc mRNA (5moU) sequence provided by TriLink Biotechnologies. We seek to replace the original

FLuc sequence with the CleanCap FLuc sequence. The original plasmid features a cytomegalovirus promoter (P_{cmv}) that drives in vivo FLuc transcription, and a simian virus 40 (SV40) polyA sequence that adds a polyA tail to FLuc mRNA and facilitates in vivo transcription termination. Additionally, the plasmid encodes an ampicillin resistance gene for plasmid maintenance in *E. coli*.

6. Our objective was to perform in vitro transcription using CleanCap co-transcription capping technology with the CleanCap FLuc mRNA (5moU) sequence. To accomplish this, the P_{T7} promoter (reverse complementary sequence: tatagtgagtcgtatta) is inserted between the P_{cmv} promoter and the gene-of-interest. The Boligo (P_{T7}-P_{cmv}-R) should contain two regions, an inner region with the sequence of the 3' end of the P_{cmv} promoter, and an outer region with the sequence of the P_{T7} promoter (**Figure S1a**).

7. In vitro transcription is a downstream application of plasmids that allows the production of mRNA from a linear DNA template. Circular plasmids can be linearized using a restriction enzyme that cuts only once, ideally immediately following the 3UTR sequence. As the new plasmid design lacked a suitable restriction enzyme recognition site, we introduced three nucleotides (atc) directly after the 3UTR sequence to create an EcoRV restriction site (gatatc) (**Figure S1a**). The Boligo (SV40 polyA-F) should contain two regions, an inner region containing the sequence of the 5' end of the SV40 polyA termination sequence and an outer region with an additional sequence (atc) to introduce the EcoRV restriction site (**Figure S1a**).

8. Insert the phosphorothioate-modified bonds into the Aoligo designs according to the principles discussed in **Figure S1a**. The actual Aoligo sequences (SV40 polyA-bff-m and P_{T7}-P_{cmv}-brr-m) are illustrated in **Figure S1a**.

9. Based on the mRNA sequencing result of CleanCap FLuc mRNA (5moU), the 5' end of the 5UTR sequence and the 3' end of the 3UTR sequence were elucidated.

10. Refer to **Figure S1b** for the principles of Aoligo and Boligo design for CleanCap TriLink FLuc. The actual Aoligo sequences (P_{T7}-TriLink FLuc-F and SV40 polyA-TriLink FLuc-R) and Boligo sequences (P_{T7}-TriLink FLuc-bff-m and SV40 polyA-TriLink FLuc-brr-m) are illustrated in **Figure S1b**.

11. Perform a visual inspection of the Aoligo and Boligo design as illustrated in **Figure S1b**.

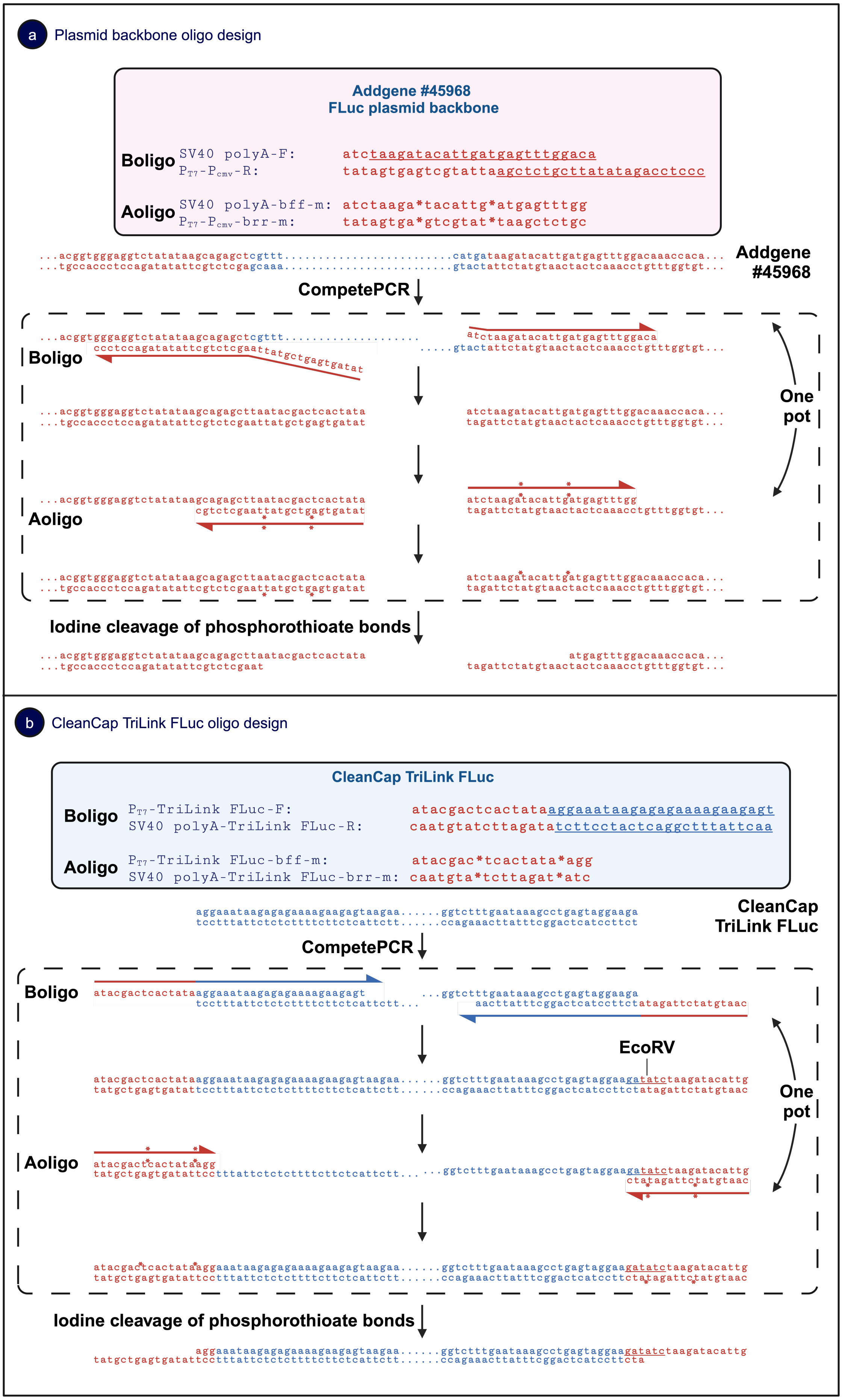
a
Plasmid backbone oligo design
Addgene #45968
FLuc plasmid backbone
Boligo
SV40 polyA-F: atctaagatacattgatgagtttggaca
PT7-Pcmv-R: tatagtgagtcgtattaagctctgcttatatagacctccc
Aoligo
SV40 polyA-bff-m: atctaaga*tacattg*atgagtttgg
PT7-Pcmv-brr-m: tatagtga*gtcgtat*taagctctgc
Addgene #45968
CompetePCR
Boligo
Aoligo
One pot
Iodine cleavage of phosphorothioate bonds
b
CleanCap TriLink FLuc oligo design
CleanCap TriLink FLuc
Boligo
PT7-TriLink FLuc-F: atacgactcactataaggaaataagagagaaaagaagagt
SV40 polyA-TriLink FLuc-R: caatgtatcttagatatcttcctactcaggctttattcaa
Aoligo
PT7-TriLink FLuc-bff-m: atacgac*tcactata*agg
SV40 polyA-TriLink FLuc-brr-m: caatgta*tcttagat*atc
CleanCap TriLink FLuc
CompetePCR
Boligo
EcoRV
Aoligo
One pot
Iodine cleavage of phosphorothioate bonds

Figure S1. Design of Aoligos and Boligos for CleanCap FLuc plasmid construction. The original plasmid (Addgene #45968) features a P_{cmv} promoter and an SV40 polyA termination sequence to facilitate transcription in mammalian cells. The top and bottom strands of the DNA sequence is illustrated in this figure. The plasmid backbone sequence is in red while the gene-of-interest that is flanked by the opposite ends of the plasmid backbone is in blue. (**a**) A P_{T7} promoter (shown as slanted) was inserted between the P_{cmv} promoter and the FLuc gene to enable in vitro transcription. Refer to Section 1.4.4 for the principles of Aoligo and Boligo design. The direction of the half arrowhead dictates how the Aoligo and Boligo should be read. For instance, the top strand is read from left to right while the bottom strand is read from right to left. The Boligo for P_{T7}-P_{cmv}-R was designed to include both the 3' end of the P_{cmv} promoter and the P_{T7} promoter. The actual Aoligo sequences (SV40 polyA-bff-m and P_{T7}-P_{cmv}-brr-m), and Boligo sequences (SV40 polyA-F and P_{T7}-P_{cmv}-R) are illustrated in **Figure S1a**. (**b**) To linearize the plasmid for in vitro transcription, an EcoRV recognition site was introduced by adding a three-nucleotide (atc) sequence (shown as slanted) after the 3' end of the 3UTR. The Boligo (SV40 polyA-F) was created to contain the SV40 polyA sequence along with an additional sequence to introduce the EcoRV site. The actual Aoligo sequences (P_{T7}-TriLink FLuc-F and SV40 polyA-TriLink FLuc-R) and Boligo sequences (P_{T7}-TriLink FLuc-bff-m and SV40 polyA-TriLink FLuc-brr-m) are illustrated in **Figure S1b**.

Reverse transcription (optional)

12. Reverse transcription is only required if the starting material (gene-of-interest) is in the form of mRNA. Proceed to the next section if you have DNA starting materials such as plasmid or gBlocks for your gene-of-interest.

13. Thaw mRNA stock (1000 ng μl^{-1}) and One*Taq* RT-PCR Kit components on ice. Thaw gene-specific reverse complementary primer (100 μM) at 25 ˚C.

14. Dilute the mRNA stock 100-fold by adding 1 μl of mRNA stock to 99 μl of Molecular Biology Grade Water. The final concentration of the mRNA solution is 10 ng μl^{-1}.

15. Add the following components to a PCR tube in the specific order: 1 μl of mRNA solution (10 ng μl^{-1}), followed by 0.3 μl of gene-specific reverse complementary primer (100 μM) and lastly topping up Molecular Biology Grade Water to a total mixture volume of 8 μl. In this protocol, the reverse gene-specific reverse complementary primer was designed to recognise CleanCap FLuc mRNA (5moU) (atcttcctactcaggctttattcaa).

16. Incubate the mRNA/primer mixture at 70 ˚C for 5 min in the thermal cycler, then immediately place the tube on ice.

17. Add the following components to the mRNA/primer mixture in the specific order: 10 μl of M-MuLV Reaction Mix, followed by 2 μl of M-MuLV Enzyme Mix. Mix thoroughly by gentle pipetting.

▲ **CRITICAL STEP** To preserve M-MuLV activity, avoid leaving the M-MuLV Enzyme Mix exposed at 25 ˚C.

18. Incubate the cDNA synthesis reaction mixture at 42 ˚C for 1 h. Inactivate the enzyme at 80 ˚C for 5 min and hold at 4 ˚C. Dilute the cDNA product mixture to 50 μl with 30 μl of Molecular Biology Grade Water.

■ **PAUSE POINT** The cDNA fragment can be used immediately in the CompetePCR workflow or stored at -20 ˚C for several years.

CompetePCR

● **TIMING ~1 h**

19. Thaw the Aoligos and Boligos at 25 ˚C. In this protocol, the following Aoligos (SV40 polyA-bff-m, P_{T7}-P_{cmv}-brr-m, P_{T7}-TriLink FLuc-bff-m and SV40 polyA-TriLink FLuc-brr-m) and Boligos (P_{T7}-TriLink FLuc-F and SV40 polyA-TriLink FLuc-R) were used.

20. Dilute the Boligos 100-fold by adding 1 µl of the original Boligo stock with 99 µl of Molecular Biological Grade Water.

21. To prepare the PCR mixture for amplifying the DNA fragment containing the plasmid backbone, add the following components to a PCR tube in the specific order at 25 ˚C: 22.8 µl of Molecular Biology Grade Water, 0.3 µl forward Aoligo (SV40 polyA-bff-m), 0.3 µl reverse Aoligo (P_{T7}-P_{cmv}-brr-m), 0.3 µl forward Boligo (SV40 polyA-F), 0.3 µl reverse Boligo (P_{T7}-P_{cmv}-R), and 1 µl of plasmid DNA template (in this protocol we used Addgene #45968 plasmid) at a final concentration of 10 ng $µl^{-1}$.

22. To prepare the PCR mixture for amplifying the DNA fragment (in this protocol we used the cDNA generated from Section "**Reverse transcription (optional)**", add the following components to a PCR tube in the specific order at 25 ˚C: 22.8 µl of Molecular Biology Grade Water, 0.3 µl forward Aoligo (P_{T7}-TriLink FLuc-bff-m), 0.3 µl reverse Aoligo (SV40 polyA-TriLink FLuc-brr-m), 0.3 µl forward diluted Boligo (P_{T7}-TriLink FLuc-F), 0.3 µl reverse diluted Boligo (SV40 polyA-TriLink FLuc-R), and 1 µl of DNA fragment at a final concentration of 1 to 10 ng $µl^{-1}$.

23. Thaw Q5 High-Fidelity 2X Master Mix on ice for 5 min or until completely thawed. Resuspend the master mix thoroughly by gently pipetting with a P200 pipette.

▲ **CRITICAL STEP** To preserve Q5 DNA polymerase activity, avoid leaving the Q5 High-Fidelity 2X Master Mix exposed at 25 ˚C. It is recommended to thaw the master mix only after preparing the PCR mixture to minimize the time spent outside the -20 °C freezer.

24. Add 25 µL of Q5 High-Fidelity 2X Master Mix to the PCR mixtures prepared in Steps 21 and 22. Mix thoroughly by gently pipetting.

25. Incubate the PCR tube in the thermal cycler using the program described in Section "**Thermal cycler setup for CompetePCR**".

■ **PAUSE POINT** The PCR product mixture may be used immediately for gel electrophoresis or stored at 4 ˚C for 24 h.

Gel electrophoresis

● **TIMING ~30 min**

26. Prepare 0.8% (wt/vol) agarose-TAE gel with SYBR Safe DNA Gel Stain setup (refer to Section "**0.8% (wt/vol) agarose-TAE gel with SYBR Safe DNA Gel Stain**") before proceeding to the gel electrophoresis workflow.
27. Add 6 µl of 6X Gel Loading Dye to the PCR product mixture. Mix thoroughly using a P200 pipette. Carefully load the samples into each well of the agarose-TAE gel using smooth and controlled pipette plunger action. In a separate well, load 10 µl of the 1-kb DNA Ladder.
28. Perform gel electrophoresis at 130 V for 25 min to resolve the DNA fragments.
29. Place the agarose-TAE gel onto a blue light transilluminator and assess whether CompetePCR yielded the expected products of the anticipated size by comparing the sample band to the 1-kb DNA ladder.
30. Excise the desired DNA band using a clean scalpel or penknife blade and transfer the excised gel slice into a 1.7-mL microcentrifuge tube.

■ **PAUSE POINT** The gel slice may be used immediately for gel extraction and cleanup or stored at 4 ˚C for 24 h.

Gel extraction and cleanup

● **TIMING ~30 min**

31. Ensure the thermomixer is pre-set to 70 ˚C at 700 r.p.m. and is ready for use.
32. Ensure the components in the GeneJET Gel Extraction Kit (Binding Buffer, Wash Buffer, and GeneJET Purification Columns) are ready for use.
33. Ensure a microcentrifuge capable of at least 20,000 x g rotational speed is ready for use.
34. Add 350 µl of Binding Buffer to each gel slice. Incubate the tube in the thermomixer at 70 ˚C with agitation at 700 r.p.m for 15 min or until the gel has completely dissolved in the Binding Buffer.
35. Transfer up to 750 µl of the solubilized gel solution to the GeneJET purification column. If the total volume exceeds 750 µl, the solution can be loaded to the column in multiple steps. After each loading, centrifuge the column at 20,000 x g for 1 min and discard the flow-through. Repeat until the entire volume of the solubilized gel solution has passed through the column membrane. Use one purification column per DNA fragment. If multiple gel slices contain the same DNA fragment, the solubilized gel solutions from these slices may be combined and loaded onto a single column.
36. Load 550 µl of Wash Buffer onto the column. Centrifuge the column at 25 ˚C for 2 min at 20,000 x g. Discard the flow-through and place the column back into the same collection tube.
37. Repeat Step 36 once.

38. Centrifuge the empty column at 25 ˚C for 2 min at 20,000 x g.

39. Transfer the column to a new 1.7-ml microcentrifuge tube. Add 25 µl of Molecular Biology Grade Water above the centre of the column membrane. Incubate at 25 ˚C for 2 min.

40. Centrifuge at 20,000 x g for 1 min at 25 ˚C.

■ **PAUSE POINT** The purified DNA fragment may be used immediately for iodine-mediated reaction or stored at -20 ˚C for several years.

Iodine-mediated reaction and cleanup

● **TIMING ~30 min**

41. Ensure the thermomixer is pre-set to 70 ˚C without agitation and is ready for use.

42. Ensure the components in the GeneJET Gel Extraction Kit (Binding Buffer, Wash Buffer, and GeneJET Purification Columns) are ready for use.

43. Ensure a microcentrifuge capable of at least 20,000 x g rotational speed is ready for use.

44. Add 5.5 µl of Tris 1.0 M solution, pH 9.0 to the purified DNA fragment sample from Step 40 and mix with gentle pipetting.

45. Add 10 µl of 30 g L^{-1} iodine solution to the DNA sample and mix with gentle pipetting.

46. Incubate the tube in the thermomixer at 70 ˚C without agitation for 5 min. **! CAUTION** Minimize deviation to incubation time to ensure efficient iodine cleavage to generate sticky ends with minimal non-specific cleavage of normal phosphate sites.

47. Dilute the sample with 250 µl Molecular Biology Grade Water.

48. Add 350 µl of Binding Buffer to the sample.

49. Repeat Steps 36 to 40 to purify the DNA fragment.

50. Determine the concentration of the DNA fragment using a NanoDrop spectrophotometer. Typical DNA fragment concentrations range from 50 to 200 ng μl^{-1}.

51. Evaluate the spectrophotometric absorbance ratios A260/A280 and A260/A230 to assess DNA purity. The A260/A280 ratio should be between 1.8 and 2.0 to ensure minimal protein contamination, while the A260/A230 ratio should fall between 2.0 and 2.2 to confirm the absence of salt or organic solvent contamination.

■ **PAUSE POINT** The purified DNA fragment may be used immediately for DNA assembly or stored at -20 ˚C for several years.

DNA assembly

● **TIMING ~30 min**

52. Prior to DNA assembly, determine the number of DNA fragments to be assembled. Use equimolar concentrations of each DNA fragment for DNA assembly. To determine the molar

concentration of each fragment, first calculate the molecular weight of the DNA fragment by inputting its sequence into a reputable online DNA molecular weight calculator. Then, determine the molar concentration by dividing the mass concentration obtained from the NanoDrop spectrophotometer (refer to Step 51) by the molecular weight of the DNA fragment. Finally, calculate the volume of each DNA fragment required for assembly, ensuring that the combined total volume of all fragments is 4 μl.

53. Add 1 μl of 10 mM magnesium chloride solution to 4 μL of the equimolar DNA fragment mixture in a PCR tube. Mix thoroughly by gentle pipetting.

54. Incubate the mixture in the thermal cycler and refer to Section "**Thermal cycler setup for DNA assembly**" for the thermal cycler setup for DNA assembly.

55. Keep the PCR tube containing the assembled DNA mixture on ice and proceed to *E. coli* transformation.

E. coli transformation

● **TIMING ~30 min**

56. Ensure the thermomixer is pre-set to 42 ˚C and ready for use.

57. Ensure the cell incubator is pre-set to 37 ˚C and ready for use.

58. Ensure that the LB agar plate containing the appropriate antibiotic is pre-warmed in the 37 ˚C incubator for at least 30 min.

59. Thaw the NEB 5-alpha Competent *E. coli* (High Efficiency) cells on ice for 5 min.

60. Mix 2 μl of the assembled DNA mixture (from Step 55) with 17 μl of *E. coli* competent cells in a pre-chilled 1.7-ml microcentrifuge tube on ice. Mix thoroughly by gentle pipetting. Incubate the tube on ice for 5 min.

61. Incubate the tube in the thermomixer at 42 ˚C for 35 s.

62. Place the tube on ice immediately for 2 min.

63. Proceed to the biological safety cabinet. Mix 100 μl of SOC Outgrowth medium with the *E. coli* competent cell solution.

64. Directly plate the cell mixture onto an LB agar plate containing the appropriate antibiotic that corresponds to the resistance gene marker present in the plasmid. For example, if the plasmid contains an ampicillin resistance gene, use an LB agar plate supplemented with ampicillin. Use an L-shaped spreader to gently spread the cell mixture across the agar surface until the plate appears dry.

65. Incubate the LB agar plate in the 37 ˚C cell incubator for 16 h. Colonies usually appear after 12 to 16 h when incubated at 37 ˚C. LB agar plates containing antibiotics may be stored at 4 ˚C in the dark for up to 1 month.

Single colony inoculation and culture

● **TIMING ~1 day**

66. Ensure the orbital shaker is pre-set to 37 ˚C at 250 r.p.m. and ready for use.

67. Proceed to the biological safety cabinet. Inoculate a single colony from the LB agar plate (refer to Step 65) into 10 ml of Terrific Broth containing 100 ng μl^{-1} of ampicillin (refer to Section "**Terrific broth containing ampicillin**"). You may pick two to three colonies per plasmid construct for screening purposes. Do not fully tighten the cap of the corning tube. Instead, screw the cap until the bottom edge of the cap coincides with the first thread of the tube to ensure consistent aeration across samples. Secure the cap in place with adhesive tape to prevent accidental loosening.

68. Incubate the culture in an orbital shaker at 37 ˚C with agitation at 250 r.p.m. for 12 to 16 h.

69. After bacterial growth is observed, add 500 μl of the cell culture to 500 μl of 60% glycerol solution in a sterile 2-mL microcentrifuge tube. Freeze the glycerol stock tube at -80 ˚C.

Plasmid extraction

● **TIMING ~30 min**

70. Ensure a microcentrifuge capable of at least 20,000 g rotational speed is ready for use.

71. Ensure the components in the ZymoPURE Plasmid Miniprep Kit are ready for use.

72. Aliquot 2 ml of the cell culture into a clean 2-ml microcentrifuge tube. Centrifuge the tube at 20,000 x g for 1 min at 25 ˚C. Discard the supernatant and retain the cell pellet.

73. Repeat Step 72 once.

74. Extract plasmid with the ZymoPURE Plasmid Miniprep Kit according to the manufacturer's protocol.

75. At the last step of Miniprep, add 25 to 50 μl Molecular Biology Grade Water above the column membrane. Incubate at 25 ˚C for 2 min.

76. Centrifuge at 20,000 x g for 1 min at 25 ˚C.

77. Determine the concentration of the plasmid using a NanoDrop spectrophotometer. Typical plasmid concentrations range from 500 to 1000 ng μl^{-1} for plasmids containing pUC19 origin of replication.

78. Evaluate the spectrophotometric absorbance ratios A260/A280 and A260/A230 to assess DNA purity. The A260/A280 ratio should be between 1.8 and 2.0 to ensure minimal protein contamination, while the A260/A230 ratio should fall between 2.0 and 2.2 to confirm the absence of salt or organic solvent contamination.

■ **PAUSE POINT** The purified plasmid can be sent for sequencing or stored at -20 ˚C for several years.

Plasmid sequencing and verification

● **TIMING ~1 day**

79. Dilute the plasmid sample according to the requirements of a third-party sequencing company. A potential third-party vendor is Azenta, which offers Nanopore whole plasmid sequencing (Plasmid-EZ).

80. Upon receipt of the sequencing data, align the FASTQ file to the plasmid sequence in silico using Benchling to assess the integrity of the plasmid sequence (**Figure S2**).

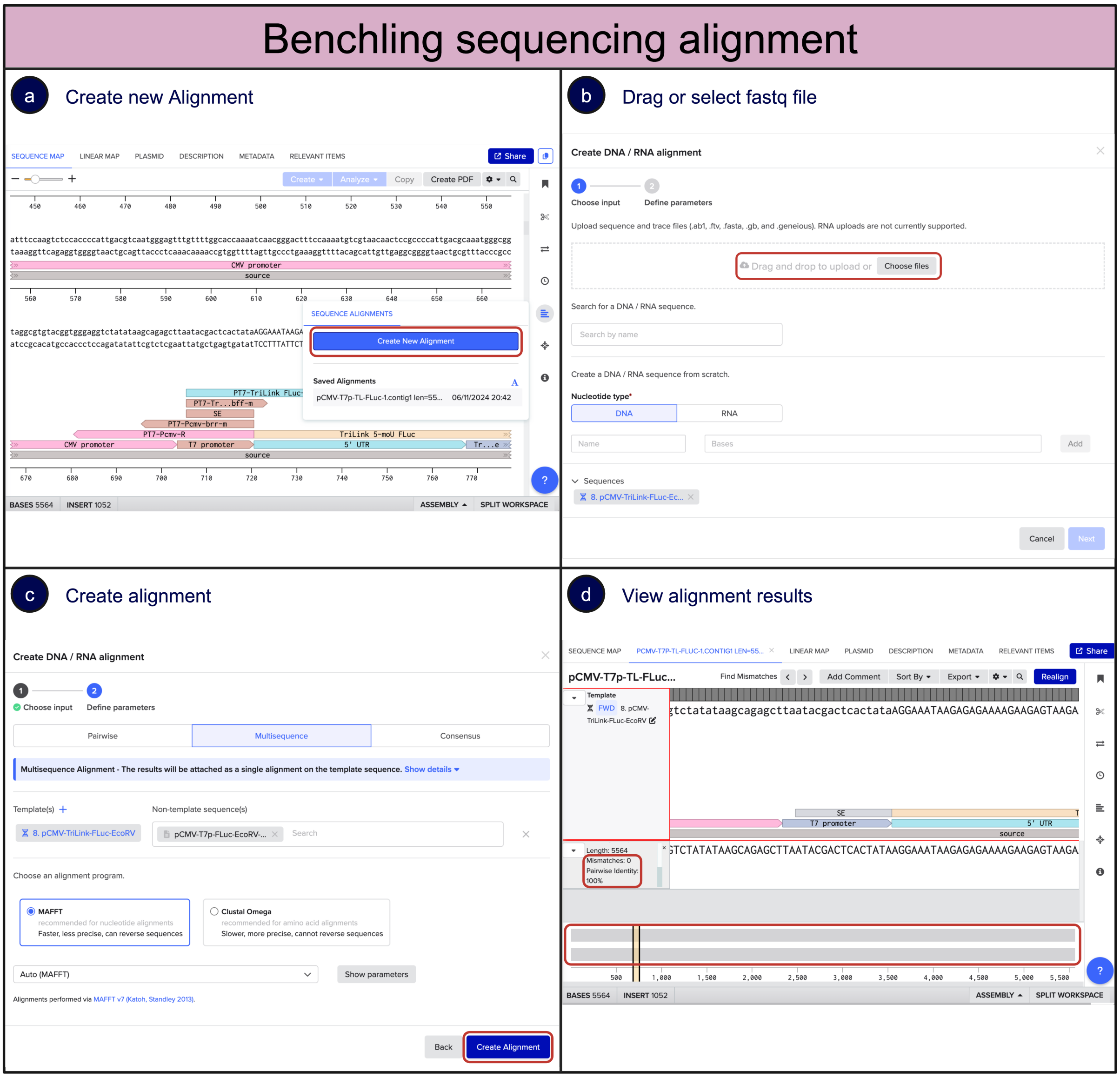

Figure S2. Benchling sequencing alignment workflow for plasmid sequence verification. (**a**) To begin the alignment process, a new alignment is created from the sequencing map of the plasmid. (**b**) The user then uploads the sequencing data, either by dragging or selecting a fastq file to provide the necessary sequence input. (**c**) Once the input is provided, a DNA/RNA alignment is created by selecting appropriate parameters, including the alignment program (e.g., MAFFT). (**d**) The alignment results are

visualized, showing the matching regions between the template plasmid and the sequencing data. The interface provides clear visualization of coverage and any discrepancies in alignment. The highlighted sections indicate specific features such as promoters and coding sequences, which can be reviewed for accuracy. This alignment workflow helps ensure plasmid integrity and sequence correctness, making it suitable for downstream applications.

Troubleshooting

Troubleshooting advice for the detailed workflow from the supplementary information can be found in this table.

Step	Problem	Possible reason	Possible solution
30	No PCR product band.<br><br>Faint/smeared PCR band.	Use of wrong oligos or DNA template.	Repeat the PCR process. Include a positive control PCR that has previously demonstrated successful amplification.
		Use of contaminated Molecular Biology Grade Water and/or PCR tube.	Use fresh Molecular Biology Grade Water and new PCR tube.<br><br>Repeat the PCR process. Include a positive control PCR that has previously demonstrated successful amplification.
		Q5 High-Fidelity 2X Master Mix was either not resuspended thoroughly prior to use, or the DNA polymerase activity has been reduced due to improper handling, such as exposing the master mix at 25 ˚C for prolonged periods.	Use new Q5 High-Fidelity 2X Master Mix.<br><br>Repeat the PCR process. Include a positive control PCR that has previously demonstrated successful amplification.
40	Low concentration of DNA fragments.	Poor gel extraction.	Consult GeneJET Gel Extraction Kit's troubleshooting instructions.
	Low A260/A230 and A260/A280 ratio.	Poor purification.	Consult GeneJET Gel Extraction Kit's troubleshooting instructions.

			In our experience, it may be possible to proceed with DNA assembly and *E. coli* transformation without affecting the plasmid concentration or sequence.
65	No colonies after *E. coli* transformation	Competent cells have low transformation efficiency due to improper handling, such as exposing competent cells at 25 ˚C for prolonged periods.	Use new competent cells.<br><br>Repeat *E. coli* transformation. Include a positive control plasmid that has previously demonstrated successful transformation. A pUC19 vector that encodes for ampicillin resistance is included in the competent cell product.
		Plasmid assembly is inefficient due to either improper preparation of DNA fragments or due to wrong oligo design. DNA fragments may fail to combine due to wrong oligo design.	Repeat *E. coli* transformation. Include a positive control DNA assembly mixture that has previously demonstrated successful transformation.
		LB agar plates were not prepared appropriately, potentially due to a higher antibiotic concentration than usual.<br><br>Use of LB agar plate that contained the wrong antibiotic.	Streak an *E. coli* strain containing a verified plasmid with the appropriate antibiotic resistance gene for the corresponding antibiotic present in the LB agar plate. Colonies should appear.
		Plasmid assembly failed due to inefficient iodine cleavage. Either the iodine solution (30 g L^{-1}) may	Ensure the iodine solution is prepared accurately at 30 g L^{-1}. Calibrate the micropipette at least

		be prepared incorrectly, or the volume of iodine solution (10 μL) per reaction was aliquoted incorrectly. Also, the iodine reaction time was not adhered to 5 mins.	every year to aliquot 10 μL of iodine solution per reaction accurately. Adhere to 5 mins for the iodine reaction time.
		Plasmid assembly failed due to challenging plasmid assembly. In theory, the complexity of plasmid assembly in general increases as the number of DNA fragments increases.	The highest number of DNA fragments that was tested for assembly was seven, with 100% assembly accuracy. If the number of fragments is much higher, consider other plasmid construction methods such as Gibson assembly.
	Bacterial lawn was observed instead of single colonies.	LB agar plates were not prepared appropriately, potentially due to a lower antibiotic concentration than usual. Alternatively, the LB agar plate may be old. Antibiotic selection pressure may be reduced.	Streak a wild-type *E. coli* strain that does not have any antibiotic resistance (consider using the competent cell) on the LB agar plate containing antibiotics. If colonies emerge, it can be concluded that the antibiotic selection pressure is reduced.<br><br>Use a new LB agar plate containing the appropriate antibiotic.<br><br>Streak an *E. coli* strain containing a verified plasmid with the appropriate antibiotic resistance gene for the corresponding antibiotic present in the LB agar plate. Colonies should appear.<br><br>Do not keep LB agar plates with antibiotics more than 1 month in the 4 ˚C refrigerator.

69	Single colony failed to grow in the cell culture.	Terrific Broth was not prepared appropriately, potentially due to a higher antibiotic concentration than usual.<br><br>Use of Terrific Broth that contained the wrong antibiotic.	Culture an *E. coli* strain containing a verified plasmid with the appropriate antibiotic resistance gene for the corresponding antibiotic present in the Terrific Broth. Single colony should grow.
78	Low concentration and yield of plasmid.	Poor plasmid extraction.	Consult ZymoPURE Plasmid Miniprep Kit's troubleshooting instructions.<br><br>Consider this positive control: culture an *E. coli* strain containing a verified plasmid with the appropriate antibiotic resistance gene for the corresponding antibiotic present in the Terrific Broth. Determine the plasmid concentration and yield of this positive control.
	Low A260/A230 and A260/A280 ratio.	Poor plasmid extraction.	Consult ZymoPURE Plasmid Miniprep Kit's troubleshooting instructions.
80	Mutations found in plasmid sequencing result.	Mutations, deletions or random nucleotide insertions are introduced during the PCR process.	Send more plasmid samples for sequencing.<br><br>If the problem persists, send the DNA fragment after PCR amplification for sequencing. If no changes are observed, the issue may be due to the plasmid encoding toxic genes, causing the cell to modify or inactivate the gene.

			Consider 30 ˚C incubation to improve plasmid stability.<br><br>You may also consider sending the original DNA template, prior to PCR amplification, for sequencing to verify the fidelity of the original template.

Full worked example

a CleanCap TriLink FLuc plasmid map

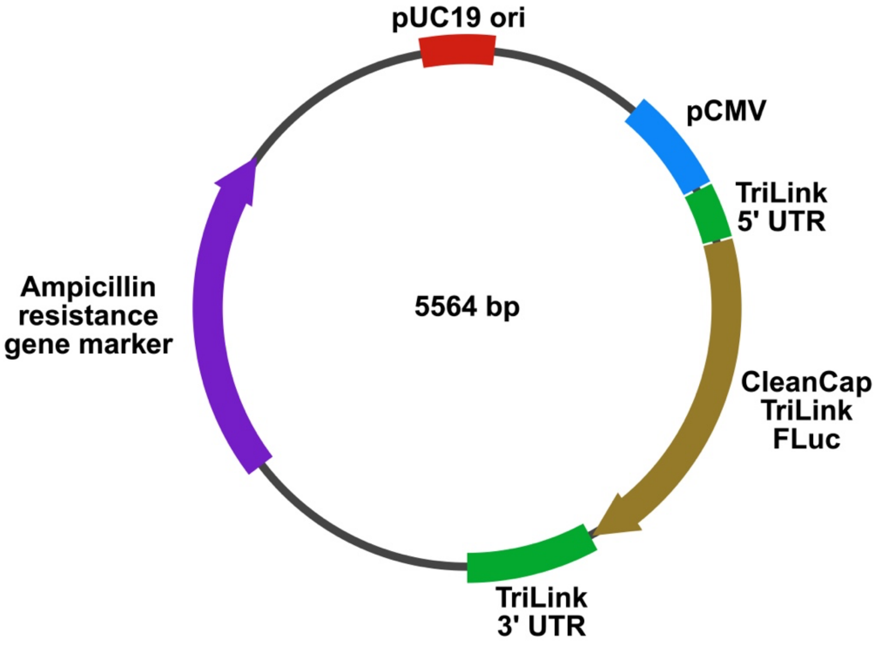

b CleanCap TriLink FLuc oligo design

CleanCap TriLink FLuc

**Boligo**	P_{T7}-TriLink FLuc-F:	atacgactcactataaggaaataagagagaaaagaagagt
	SV40 polyA-TriLink FLuc-R:	caatgtatcttagatatcttcctactcaggctttattcaa
**Aoligo**	P_{T7}-TriLink FLuc-bff-m:	atacgac*tcactata*agg
	SV40 polyA-TriLink FLuc-brr-m:	caatgta*tcttagat*atc

c Expected gel photo

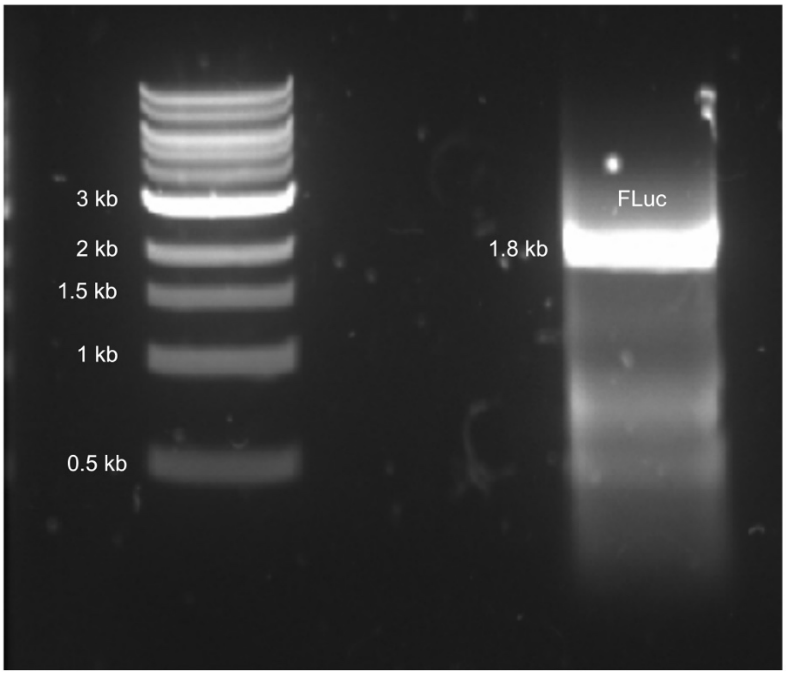

Figure S3. Full worked example of assembling CleanCap TriLink FLuc plasmid. (**a**) Plasmid map of CleanCap TriLink FLuc plasmid. (**b**) Oligonucleotide sequences to amplify CleanCap TriLink FLuc DNA fragment. (**c**) Expected gel photo of CleanCap TriLink FLuc DNA fragment after PCR.

Table S1. Comparing between different plasmid assembly methods.

S/N	Plasmid	Size (kb)	No. of DNA fragments	Genes	Gene length (kb)	GC%	Plasmid assembly efficiency (%)			Reference
							Gibson assembly	Restriction enzyme	Phosphorothioate -modified	
1.	nupG	3.2	5	AR	1.1	51.7	66.7	100	100	1
				RO	0.9	53.6				
				gRNA	0.2	38.0				
				UHSa	0.5	48.3				
				DHSa	0.5	53.4				
2.	TPP7	6.4	7	AR	1.1	51.7	n.d.	100	100	
				RO	0.9	53.6				
				pLac	0.1	39.5				
				aroG	1.2	51.5				
				tyrA	1.2	50.5				
				t7t	0.2	55.0				
				lacI	1.7	54.0				

Table S2. Representative example of plasmids constructed using phosphorothioate-modified oligonucleotides.

S/N	Plasmid	Size (kb)	No. of DNA fragments	Reference
1.	S-IAA-PAC	8.7	2	[2]
	S-DEF-IAA-PAC	11.3	3	
	S-R-DEF-IAA-PAC	13.1	4	
	S-R-DEF-GH-IAA-PAC	15.6	5	
	S-R-DEF-GH-ISC-IAA-PAC	21.6	6	
2.	nupG	3.2	5	[1]
	TPP1	6.7	6	
	TPP7	6.44	7	
3.	INT_GFPsp (Addgene #212144)	13.1	2	[3]
	INT_Cargo_T7_CbFDH (Addgene #212140)	5.2	3	
	pXU01 (Addgene #212151)	5.1	4	
4.	pGU02	11.1	5	[4]
	pGU03	7.6	5	
	pGU04	7.5	4	

References

1. Ma, X., Liang, H., Cui, X., Liu, Y., Lu, H., Ning, W., Poon, N.Y., Ho, B., and Zhou, K. (2019). A standard for near-scarless plasmid construction using reusable DNA parts. Nature Communications *10*. 10.1038/s41467-019-11263-0.
2. Zou, R., Zhou, K., Stephanopoulos, G., and Too, H.P. (2013). Combinatorial Engineering of 1-Deoxy-D-Xylulose 5-Phosphate Pathway Using Cross-Lapping In Vitro Assembly (CLIVA) Method. PLoS ONE *8*. 10.1371/journal.pone.0079557.
3. Fung, V., Xiao, Y., Tan, Z.J.D., Ma, X., Zhou, J.F.J., Panda, S., Yan, N., and Zhou, K. (2022). Producing aromatic amino acid from corn husk by using polyols as intermediates. Biomaterials *287*. 10.1016/j.biomaterials.2022.121661.
4. Fung Kin Yuen, V., Tan Zhi Jun, D., and Zhou, K. (2023). Improving glycine utilization in Escherichia coli. Biochemical Engineering Journal *193*. 10.1016/j.bej.2023.108834.
